# Care Experiences and Expectations of Older Sexual Minority Adults

**DOI:** 10.1093/geroni/igab046.3364

**Published:** 2021-12-17

**Authors:** Mekiayla Singleton, Susan Enguidanos

**Affiliations:** University of Southern California, Los Angeles, California, United States

## Abstract

Sexual minority (SM) adults have unique care needs and experiences, partially because they receive and give care by and to “chosen family”. This study examines the care experiences and expectations of diverse SM adults. Using data from the 2018 AARP Survey “Maintaining Dignity: Understanding and Responding to the Challenges Facing Older LGBT Americans,” logistic and ordinal regressions were conducted to examine associations with care experiences (i.e., provided caregiving and received caregiving) and care expectations (i.e., likelihood of having to provide care and need care) among SM respondents. Gender was highly associated with care experiences, with female respondents being 70% and 74% more likely to have provided caregiving [OR:1.71, SE=.26; p<0.001] and received caregiving [OR:1.74, SE=.22; p< 0.001]. Relationship status was significantly associated with care expectations, with those who were married/civil union/domestic being 4 times [OR:4.0, SE=.52; p<0.001] and those in a relationship being 3 times [OR:3.3, SE=.51; p<0.001] more likely to expect that they will provide care in the future. Those same respondents had a 64% [OR:1.64, SE=.21, p<0.001] and 55% [OR:1.55, SE=.23, p<0.01] greater odds of reporting being “very likely” that they will need care in the future. Additionally, older age, being a racial minority, having higher education, and being employed were significantly and positively associated with care experiences and expectations. These findings provide a deeper insight into how SM individuals of different backgrounds experience and anticipate different aspects of caregiving. Moreover, we will discuss how our findings compare to non-SM individuals and implications of these findings.

